# Urogenital abnormalities in men exposed to diethylstilbestrol *in utero*: a cohort study

**DOI:** 10.1186/1476-069X-8-37

**Published:** 2009-08-18

**Authors:** Julie R Palmer, Arthur L Herbst, Kenneth L Noller, Deborah A Boggs, Rebecca Troisi, Linda Titus-Ernstoff, Elizabeth E Hatch, Lauren A Wise, William C Strohsnitter, Robert N Hoover

**Affiliations:** 1 Slone Epidemiology Center of Boston University, Boston, MA, USA; 2 Department of Obstetrics and Gynecology, University of Chicago, Chicago, IL, USA; 3 Department of Obstetrics and Gynecology, New England Medical Center, Boston, MA, USA; 4 Division of Cancer Epidemiology and Genetics, National Cancer Institute, Bethesda, MD, USA; 5 Departments of Community and Family Medicine and of Pediatrics, Dartmouth Medical School, Lebanon, NH, USA; 6 Department of Epidemiology, Boston University School of Public Health, Boston, MA, USA

## Abstract

**Background:**

Diethylstilbestrol (DES), a synthetic estrogen widely prescribed to pregnant women during the 1940s-70s, has been shown to cause reproductive problems in the daughters. Studies of prenatally-exposed males have yielded conflicting results.

**Methods:**

In data from a collaborative follow-up of three U.S. cohorts of DES-exposed sons, we examined the relation of prenatal DES exposure to occurrence of male urogenital abnormalities. Exposure status was determined through review of prenatal records. Mailed questionnaires (1994, 1997, 2001) asked about specified abnormalities of the urogenital tract. Risk ratios (RR) were estimated by Cox regression with constant time at risk and control for year of birth.

**Results:**

Prenatal DES exposure was not associated with varicocele, structural abnormalities of the penis, urethral stenosis, benign prostatic hypertrophy, or inflammation/infection of the prostate, urethra, or epididymus. However, RRs were 1.9 (95% confidence interval 1.1-3.4) for cryptorchidism, 2.5 (1.5-4.3) for epididymal cyst, and 2.4 (1.5-4.4) for testicular inflammation/infection. Stronger associations were observed for DES exposure that began before the 11^th ^week of pregnancy: RRs were 2.9 (1.6-5.2) for cryptorchidism, 3.5 (2.0-6.0) for epididymal cyst, and 3.0 (1.7-5.4) for inflammation/infection of testes.

**Conclusion:**

These results indicate that prenatal exposure to DES increases risk of male urogenital abnormalities and that the association is strongest for exposure that occurs early in gestation. The findings support the hypothesis that endocrine disrupting chemicals may be a cause of the increased prevalence of cryptorchidism that has been seen in recent years.

## Background

Diethylstilbestrol (DES), a synthetic estrogen that was widely prescribed to pregnant women during the 1940s to 1970s, has been associated with an increased prevalence of abnormalities of the reproductive tract in women who were exposed in utero [1]. Whether there are similar effects in the exposed sons is still unclear and only a few studies have appreciable numbers. Two studies found a higher prevalence of genital tract abnormalities among exposed men [2-5]. A third study, however, reported no association between prenatal DES exposure and genital tract abnormalities [6]. In a continuation of follow-up of U.S. prospective cohorts of DES-exposed and unexposed sons, we combined data from both of the previous prospective studies discussed above and a third, previously unexplored, cohort of exposed and unexposed men to re-examine this question and investigate modifying effects of the timing and dose of DES exposure.

## Methods

### Study design

In 1994, a collaborative follow-up study of all existing U.S. cohorts of DES-exposed men and women was initiated by the U.S. National Cancer Institute. Three cohorts of men exposed to DES in utero were included. One comprised the sons of women who participated in a randomized trial of DES at the University of Chicago in the early 1950s. In the 1970s, the current addresses of these young men were determined, after which they were followed periodically with examinations and questionnaires [7]. A second group included the sons of women who had been treated in a private infertility practice near Boston between 1943 and 1975; they have been followed by questionnaire since 1975. The third group included men whose mothers were identified as DES-exposed or unexposed by systematic review of prenatal records at the Mayo Clinic for the period from 1939 to 1962; questionnaire follow-up of this group began in 1982 [8]. The study protocol for the present collaborative study has been approved by institutional review boards of all participating institutions and the National Cancer Institute.

From the three original cohorts, 229 men had never been located, 136 had already died, and 463 had declined participation. Thus, 3067 men (1638 exposed, 1429 unexposed) were eligible for the collaborative follow-up study. All eligible men were contacted by mailed questionnaires in 1994 and asked to participate in the collaborative study. Questionnaires were obtained from 1156 exposed men (71 percent) and 984 unexposed men (69 percent). Of them, 91 percent of exposed and 94 percent of unexposed completed a 1997 follow-up questionnaire, and 91 percent each of exposed and unexposed completed a 2001 follow-up questionnaire.

For all three cohorts, DES exposure status was determined by review of mothers' medical records. Information on cumulative DES dose was available for 52 percent of exposed men. Five grams was used as a cutpoint for "high" versus "low" dose because the distribution of cumulative dose was bimodal, with peaks at around 2 and 12 grams. Timing of first exposure to DES was available for 90 percent of exposed men. A cut-point of 11 weeks gestational age was used because the male genitalia are most susceptible to teratogens in this early period (the first nine weeks of gestation) [9].

The 1994, 1997, and 2001 questionnaires included questions on demographic and lifestyle factors, reproductive history, and medical history. Respondents were asked if they had ever been diagnosed with any of the following urogenital abnormalities: undescended testis, epididymal cyst, varicocele, structural abnormality of penis, urethral abnormality, or any other condition of the genital tract. They were also asked if they had ever been diagnosed with infection or inflammation of the prostate, urethra, testicle, or epididymal tube.

### Data analysis

We used Cox regression with constant time at risk to estimate risk ratios (RRs) and 95 percent confidence (CI) intervals for the association of DES exposure with urogenital outcomes [10]. All analyses were adjusted for year of birth and cohort. SAS statistical software (version 9.1) was used for all analyses.

## Results

Most men, both exposed (78 percent) and unexposed (78 percent), reported having at least one physical examination in the 5 years prior to 1994, the year of the baseline questionnaire. Similar proportions of exposed (18 percent) and unexposed (17 percent) reported having had a urologic examination. The age distributions of the exposed and unexposed cohorts were similar: in 1994, the median age was 43 in the exposed cohort and 42 in the unexposed cohort.

Varicocele, urethral stenosis, and structural abnormalities of the penis, including hypospadias, were not associated with prenatal DES exposure (Table 1). In contrast, the RRs for exposed versus unexposed men were 1.9 (95 percent CI 1.1-3.4) for cryptorchidism and 2.5 (95 percent CI 1.5-4.3) for epididymal cyst. Only four men reported both conditions. Elevated RRs for these outcomes were observed in all three cohorts (data not shown).

**Table 1:** DES exposure in relation to urogenital abnormalities

	DES-Exposed (N = 1197)	Unexposed (N = 1038)		
				
	Cases	Cases	Risk ratio*	95% Confidence interval
Urogenital abnormalities				
Cryptorchidism	38	17	1.9	1.1-3.4
Epididymal cyst	55	19	2.5	1.5-4.3
Varicocele	63	61	0.9	0.6-1.3
Abnormality of penis†	9	7	1.1	0.4-3.0
Urethral stenosis	14	9	1.3	0.6-3.1
Inflammation of:				
Prostate	81	61	1.1	0.8-1.6
Urethra	48	28	1.5	0.9-2.4
Testes	49	17	2.5	1.5-4.4
Epididymis	39	34	1.0	0.6-1.6
Benign prostatic hypertrophy	19	14	1.2	0.6-2.3

*Risk ratios adjusted for year of birth and cohort.

†Including hypospadias.

DES exposure was not associated with the occurrence of benign prostatic hypertrophy or with inflammation or infection of the urethra, epididymus, or prostate. There was a positive association with inflammation or infection of the testes (RR = 2.5, 95 percent CI 1.5-4.4).

As shown in Table 2, associations of DES exposure with cryptorchidism, epididymal cyst, and infection/inflammation of the testes were stronger for men who were first exposed before the 11^th ^week of gestation than for men first exposed later in gestation. Stronger associations were also observed for cumulative exposures of five or more grams of DES.

**Table 2:** Timing and dose of DES exposure in relation to urogenital abnormalities

	Cases/Total	Risk ratio*	95% Confidence interval
Cryptorchidism			
Timing of DES exposure			
Began after 11^th ^wk	8/451	1.1	0.5-2.5
Began before 11^th ^wk	29/625	2.9	1.6-5.2
			
Cumulative dose < 5 g	7/231	1.9	0.8-4.5
Cumulative dose ≥ 5 g	20/389	3.2	1.7-6.0
			
Epididymal cyst			
Timing of DES exposure			
Began after 11^th ^wk	17/451	2.0	1.0-3.8
Began before 11^th ^wk	38/625	3.5	2.0-6.0
			
Cumulative dose < 5 g	11/231	2.7	1.3-5.7
Cumulative dose ≥ 5 g	28/389	4.0	2.3-7.1
			
Inflammation of testes			
Timing of DES exposure			
Began after 11^th ^wk	16/451	2.1	1.1-4.2
Began before 11^th ^wk	30/625	3.0	1.7-5.4
			
Cumulative dose < 5 g	9/231	2.0	1.1-3.8
Cumulative dose ≥ 5 g	23/389	3.2	1.7-5.8

*Risk ratios adjusted for year of birth and cohort.

A previous investigation of participants in the Mayo Clinic study reported null findings regarding a possible association of prenatal DES exposure with risk of epididymal cyst, cryptorchidism, or other urogenital abnormalities [6]. DES doses were generally low in the population served by the Mayo Clinic, and the timing of first exposure varied greatly [8]. We carried out separate analyses among participants from the Mayo cohort to assess whether timing of DES exposure would explain the earlier results. Whereas there were no statistically significant associations for DES exposure overall, epididymal cyst and cryptorchidism were significantly associated with prenatal DES exposure that began before the 11^th ^week of gestation (Table 3). Cumulative dose information was available for only 20 percent of Mayo participants, and the cumulative dose was 5 grams or higher for only one case of cryptorchidism and one case of epididymal cyst.

**Table 3:** Results for cohort of men born at the Mayo Clinic

	Cases/Total	Risk ratio*	95% Confidence interval
Cryptorchidism			
Any DES exposure	17/675	1.7	0.7-3.8
Began after 11^th ^week	7/347	1.3	0.5-3.5
Began before 11^th ^week	9/252	2.4	1.0-6.2
			
Epididymal cyst			
Any DES exposure	21/674	1.5	0.7-3.1
Began after 11^th ^week	10/347	1.3	0.5-3.2
Began before 11^th ^week	11/252	2.2	1.0-5.0

*Risk ratios adjusted for year of birth.

## Discussion

Men who were exposed to DES in utero had an increased prevalence of cryptorchidism, epididymal cysts, and inflammation/infection of the testes. The associations were strongest for exposure before the 11^th ^week of gestation and for a cumulative dose of DES of 5 grams or more. Because nearly all women who receive a cumulative dose of 5 grams or greater had begun taking DES before the 11^th ^week of gestation, it was not possible to determine definitively which of these factors was more important.

Studies of rats [11] and mice [12] given DES during pregnancy found that the male offspring had a higher than expected prevalence of cryptorchidism and epididymal cysts. The occurrence of epididymal cysts may be due to the persistence of mullerian remnants. It has been hypothesized that exogenous female sex hormones, acting transplacentally, might interfere with the action of mullerian inhibiting factor [13]. Normal descent of the testes is, at least in part, under hormonal control [14]. The first stage, transabdominal descent, occurs during the first trimester of pregnancy under the influence of insulin-like factor 3, which is inhibited by 17 β-estradiol in mice embryonic cells. The second stage, inguinal-scrotal descent, is induced by androgens and usually occurs during weeks 26-35. In a case-control study of boys with cryptorchidism, Bernstein et al. found that the mothers of cases had higher levels of free and albumin-bound estradiol during pregnancy as compared with controls, but there was no difference in total estradiol concentration [15]. A similar study found higher levels of total estradiol in the mothers of cases overall but did not find a difference for levels during the first trimester of pregnancy [16]. A third study also found no differences between cases and controls in mother's total estradiol in the first trimester of pregnancy [17]. DES is a more potent estrogen than estradiol and may have other chemical effects as well.

An examination study of 308 DES-exposed and 307 unexposed sons of women who participated in the University of Chicago randomized trial indicated a higher prevalence of epididymal cysts and of hypoplastic testes in exposed relative to unexposed men [4]. Sixteen years later, a questionnaire study of the same cohort of men also indicated an association between DES exposure and these outcomes [5]. This study found the observed associations to be stronger among men whose mothers DES treatment had started less than 11 weeks after their last menstrual period as compared with men whose mothers had begun taking DES later in the pregnancy. Subsequent to the examination study from the Chicago cohort, a separate examination study was carried out in 265 exposed and 274 unexposed men who had been delivered at the Mayo Clinic between 1939 and 1962 [6]. Contrary to findings from the University of Chicago study, the Mayo Clinic study reported no association between prenatal DES exposure and epididymal cyst or anomalies of the testes (including cryptorchidism) [6].

The present findings help to resolve previous discrepancies in results. The weaker association observed in the Mayo Clinic cohort in the present study and the conflicting results from earlier studies can be explained by differences in timing and cumulative dose of DES. Women treated at the Mayo Clinic typically took DES for a few weeks to a few months and their median cumulative dose was 720 mg [6]. For the University of Chicago trial, women were instructed to begin taking DES at entry into the trial, usually within the first trimester of pregnancy, and continue with increasing doses until a week or two before delivery [7]. The typical cumulative dose for this cohort was around 11 grams. The third cohort making up the present study consisted of offspring of women from an infertility practice in which the usual protocol was to take DES throughout the pregnancy, resulting in a high cumulative dose. Therefore, the Mayo Clinic cohort differs from the other cohorts in two major ways: only 48 percent were exposed before the 11^th ^week of gestation (as shown in Table 3) and very few were exposed to 5 grams or more. When the analysis of Mayo Clinic sons was stratified on timing of first exposure, it became clear that there was indeed an association of DES exposure with occurrence of cryptorchidism and epididymal cysts if exposure began before the 11^th ^week of pregnancy, and that the magnitude of the association was similar to that observed in the overall study data.

Interestingly, timing of in utero exposure to DES has also been shown to be a predictive factor for structural anomalies of the lower genital tract in exposed daughters. In a study of structural anomalies of the cervix and vagina in DES-exposed daughters, investigators found a higher prevalence of anomalies (44 percent) in women who were prenatally exposed to DES before the 15^th ^week of gestation, compared to prevalences of 22 percent for weeks 15-22 and 5 percent for weeks 23 and greater [18].

The chief limitation of our study is reliance on self-reports for all outcomes. It is certainly possible that urogenital abnormalities may be more likely to come to diagnosis among DES-exposed men, and that DES-exposed men may be more likely than unexposed men to remember and report such abnormalities or inflammation. The fact that associations were observed for only three of ten conditions suggests an explanation other than reporting bias. Furthermore, the stronger associations observed for exposure that began early in pregnancy and for high cumulative dose of DES would not be explained by reporting bias since it is highly unlikely that there would be differential reporting of outcomes according to dose or timing.

The association with inflammation/infection of the testes may be a chance finding since we examined a number of outcomes and did not have an a priori hypothesis for finding an association with this endpoint. In addition, we did not have information on the specific conditions or when they occurred. It is possible that minimal structural abnormalities, such as minor obstructions, which may be associated with prenatal DES exposure, could explain this association. Further follow-up is needed to establish whether the association with inflammation/infection of the testes is due to chance or is causal. It is reassuring, however, to find no evidence of an increase risk of benign prostatic hypertrophy in the DES-exposed group.

Study strengths include the unbiased selection of exposed and unexposed groups, medical record documentation of exposure status, and a large sample size. The size of the study population permitted a meaningful analysis by timing and dose, which helped to explain previously discrepant findings.

## Conclusion

The present results indicate that DES exposed sons have a higher occurrence of cryptorchidism and epididymal cysts than unexposed sons, and that the increased risk is related to timing and dose. Fortunately, it has already been shown that prenatal DES exposure in men does not materially affect fertility, even in men with urogenital anomalies [5,19,20]. From a public health viewpoint, the importance of these findings lies in their applicability to the question of whether environmental factors acting as endocrine disruptors may have a detrimental effect on the male reproductive system. The prevalence of cryptorchidism appears to have increased in recent years and it has been hypothesized that it is caused by a combination of genetic and environmental factors, including endocrine disrupting chemicals [21-23]. Under this hypothesis, the increased prevalence of cryptorchidism and related conditions may be related to increased estrogen exposure in utero. In animal studies, exogenous estrogens have been shown to cause disorders of genital development [24]. Our confirmation of an increased risk of these conditions in men prenatally exposed to high doses of DES lends credence to this hypothesis. As cohort participants age, it will become possible to investigate whether DES-exposed men have an increased risk of conditions such as benign prostatic hypertrophy and prostate cancer, which occur more often in older men.

## List of abbreviations

DES: Diethystilbestrol; RR: risk ratio; CI: confidence interval.

## Competing interests

The authors declare that they have no competing interests.

## Authors' contributions

JRP participated in the design of the study, acquisition of data, and interpretation of results, and drafted the manuscript.

ALH, KLN, RT, LTE, EEH, and RNH participated in the design of the study, acquisition of data, interpretation of results, and had critical input into the manuscript.

DAB and LAW participated in data analysis, interpretation of results, and had critical input into the manuscript.

WCS participated in acquisition of data, interpretation of results, and had critical input into the manuscript.
